# Daily spirometry in an acute exacerbation of adult cystic fibrosis patients

**DOI:** 10.1177/1479972317743756

**Published:** 2017-11-28

**Authors:** Michael J Stephen, Alex Long, Chad Bonsall, Jeffrey B Hoag, Smita Shah, Dorothy Bisberg, Douglas Holsclaw, Laurie Varlotta, Stan Fiel, Doantrang Du, Robert Zanni, Denis Hadjiliadis

**Affiliations:** 1 Division of Pulmonary and Critical Care, Drexel University School of Medicine, Philadelphia, PA, USA; 2 Drexel University School of Public Health, Philadelphia, PA, USA; 3 Division of Pulmonary and Critical Care, University of Pennsylvania, Philadelphia, PA, USA; 4 Barnabas Health Medical Group, Saint Barnabas Medical Center, Pediatric Specialty Center, West Orange, NJ, USA; 5 St Chrisophter’s Hospital for Children, Philadelphia, PA, USA; 6 Atlantic Health System, Morristown, NJ, USA; 7 Monmouth Medical Center, Long Branch, NJ, USA

**Keywords:** Cystic fibrosis, acute exacerbation, home spirometry, CFQ-R, intravenous antibiotics

## Abstract

To help answer the question of length of intravenous antibiotics during an acute exacerbation of cystic fibrosis (CF), we had subjects to follow daily home spirometry while on intravenous antibiotics. CF patients, 18 and older, with an acute exacerbation requiring intravenous antibiotics had a daily FEV1. The average time to a 10% increase over their initial sick FEV1 was calculated, as well as the time to a new baseline. A total of 25 subjects completed the study. Ten of the 25 subjects did not have a sustainable 10% increase in FEV1. Of the 15 subjects with a sustainable 10% increase in FEV1, it took 5.2 days (±4.5) after day 1, while a new baseline was achieved on average at 6.6 days (±4.8) after day 1. Given the wide range of time to a 10% improvement and new baseline, it is recommended there should be flexibility in length of intravenous antibiotics in CF, not by a preset number.

## Introduction

Acute exacerbations in cystic fibrosis (CF) are a leading cause of morbidity and mortality. An acute exacerbation in two separate studies was negatively correlated with 2- and 5-year survival rates.^1,2^ Acute exacerbations are also associated with lower lung function later in life, increased health-care costs, and decreased quality of life.^3–5^ Many subjects also fail to get back to their pre-exacerbation baseline for spirometry.^6,7^ Episodes of acute exacerbation affect not only the lungs but also psychological well-being.^8,9^ Exacerbations also occur more frequently with increasing age and declining lung function, creating a downward cycle as subjects get sicker.^10^

The underlying causes for an acute exacerbation are not well described, and although guidelines exist, there is no universally agreed upon standardized treatment regimen. Randomized trials demonstrating the efficacy of intravenous antibiotics have been small but have shown an improvement in the bacterial load in the airways, as well as an improvement in the FEV1 and symptoms.^11,12^

Despite their widespread use, the optimal length of intravenous therapy is unclear. A recent Cochrane review, as well as the 2009 CF foundation guidelines on the treatment of an acute exacerbation, identified no trials that would be helpful to answer this question.^13,14^ Several observational studies using spirometry have yielded conflicting results, with some advocating shorter courses of antibiotics than the standard 14 days, and others for an individualized approach.^15–17^

Pulmonary function testing is one surrogate marker to follow lung improvement to inform us when the benefits of intravenous antibiotics occur and also potentially when they could be stopped. FEV1 is an outcome measure followed commonly in clinical trials for medication efficacy and has correlated with mortality in CF.^18,19^ In an effort to understand the effects of intravenous antibiotics, we undertook a prospective observational cohort study of daily spirometry in CF subjects, 18 and older, during an acute exacerbation.

## Materials and methods

### Study design

CF subjects who were deemed by their physician to have a severe acute exacerbation requiring intravenous antibiotics were eligible for participation. Baseline characteristics were collected, and subjects were given a handheld home spirometer along with instructions for usage (PiKo-6^®^; nSpire Health, Inc., Longmont, Colorado USA). Subjects were asked to check their FEV1 every morning after airway clearance. They performed daily spirometry during the time they were on intravenous antibiotics. This data were not used by clinicians to tailor antibiotic length. Baseline characteristics were collected as summarized in Table 1, including their baseline FEV1 based on a prior well visit in the last 6 months before their acute exacerbation. Each patient also completed a CF Questionnaire-Revised (CFQ-R) at day 1 and weekly during their exacerbation. Institutional Review Board approval was obtained at all participating sites prior to enrollment.

**Table 1.** Baseline characteristics.^a^

	Total (*N* = 25)
Age (years)	28.9 ± 8.4
Female, *n* (%)	19 (76)
Baseline BMI (kg/m^2^)	22.71 ± 6
FEV1% predicted (baseline)	56 ± 19
Diabetes at baseline, *n* (%)	6 (24)
*Pseudomonas aeruginosa*, *n* (%)	22 (88)
Methicillin-resistant *Staphylococcus aureus*, *n* (%)	8 (32)
Pancreatic insufficiency, *n* (%)	22 (88)
Sweat test (mM)	98.8 ± 24
Genotype	
DF508 homozygous, *n* (%)	10 (40)
DF508 heterozygous, *n* (%)	11 (44)
Other, *n* (%)	4 (16)
Length of intravenous antibiotics	18.2 ± 6.8
Average percent days spent inpatient	51 ± 34
Use of inhaled antibiotics during exacerbation, *n* (%)	7 (28)
DNAse usage, *n* (%)	24 (96)
Average number of intravenous antibiotics	2.2 ± 0.57

BMI: body mass index; SD: standard deviation.

^a^All data are mean ± SD unless specified to be count (%).

### Sample

Patients, 18 and over, from six centers from the Mid-Atlantic region recruited subjects for the study: Drexel University adult, University of Pennsylvania adult, Morristown Medical Center adult, Monmouth Medical Center adult and pediatrics, St Christopher’s Hospital for Children pediatrics, and Barnabas Health pediatrics. The Drexel University Adult center served as the lead center for data collection and analysis. Subjects were enrolled between January 1, 2013 and August 1, 2015.

### End points and statistical analysis

Two primary end points were followed on each patient. The first was the time it took to achieve a sustainable increase in the FEV1. To calculate this, an initial sick baseline was first documented, which was defined as the FEV1 on the first day of the exacerbation. The time it took to achieve a sustainable increase over their initial sick baseline was defined as the first day of three consecutive days where the FEV1 increased by at least 10% over their initial sick baseline.

The second calculation was the time it took to achieve a new baseline. The new baseline was defined as the average of the subjects’ final three values of their FEV1 while on intravenous antibiotics. The day on which the subject first got within 5% of this new baseline was then determined. Subjects were then subdivided based on whether they reached 5% of their baseline value only once during their treatment course or if once they achieved a value within 5% of their new baseline, it was then sustained.

A logistic regression to estimate the effect that baseline percent FEV1 had on the probability that a subject had a sustainable increase in their FEV1 measurement, as defined above, was performed both with the subject’s baseline percent FEV1 as a continuous predictor and as a categorical variable with baseline percent FEV1 dichotomized as less than 50% or greater than or equal to 50%.

Finally, with regard to lung function, a ratio of the subject’s sick baseline on day 1 of their exacerbation was calculated to their prior well baseline. The prior well baseline was a value in the previous 6 months when the patient was known to be in good health. This was provided by the treating team based on chart review. The prior well baseline was also compared with the new baseline in a ratio to determine if subjects were able to get back to their prior lung function. A Wilcoxon Signed Rank Test was then performed to compare the sick baseline to the old baseline, as well as the new baseline.

## Results

Baseline characteristic is summarized in Table 1. A total of 25 subjects were recruited into the study. The mean age was 28.9 years, and 76% of the subjects were women. Ten subjects (40%) were homozygous for delF508, and 11 (44%) were heterozygous for delF508. The mean body mass index was 22.7. Pseudomonas colonization was present in 88% of subjects, and the same percent were pancreatic insufficient. There were no *Burkholderia cepacia* subjects enrolled, whereas subjects with Methicillin-resistant *Staphylococcus aureus* constituted 32% of the population. The average FEV1 was 56.2% of predicted, suggesting a sicker than average adult cohort. The average length of intravenous antibiotics was 18 days.

**Figure 1.**
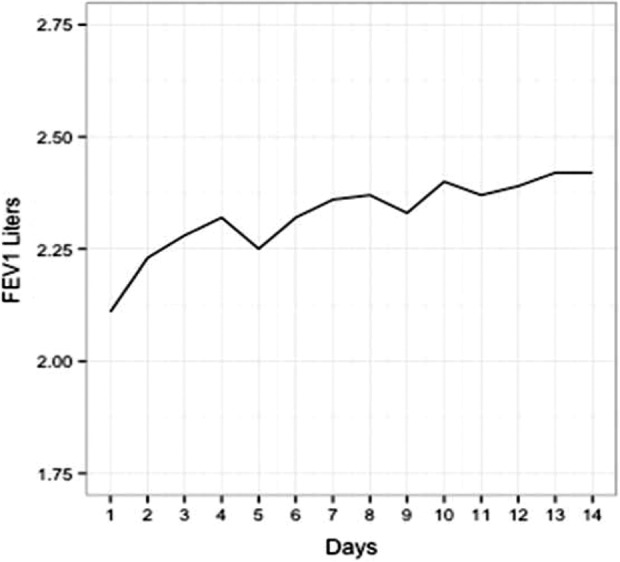
FEV1 graph for a single subject with a sustainable increase. This subject had a prior well baseline of 2.55 (89% predicted) and started IV antibiotics with a sick baseline on day 1 of 2.11. This gives the subject a ratio of well to sick baseline of 0.83. The new baseline, defined as the average of the final three FEV1 values, was 2.41. The subject achieved a sustainable 10% increase from their sick baseline of 2.11 on day 6 and got within 5% of their sick baseline and sustained this effort on day 6. Their new baseline to old baseline was 0.95, indicating they did not get back to their prior well baseline at the end of intravenous antibiotics.

**Figure 2.**
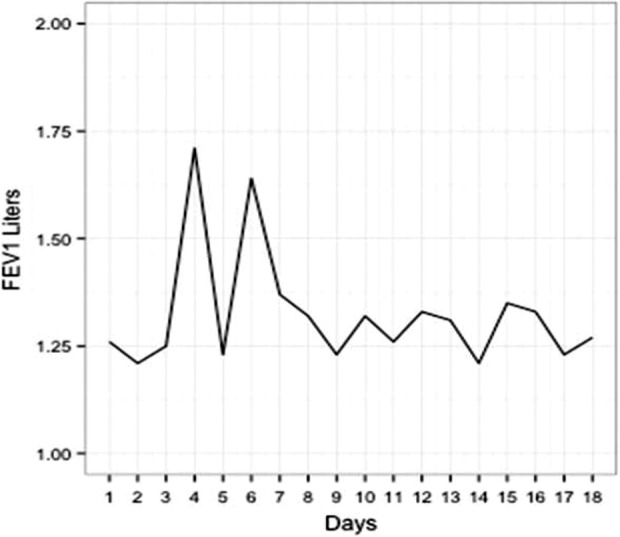
FEV1 graph for a single subject without a sustainable increase. This subject had a prior well baseline of 1.36 (47% predicted) and a sick baseline of 1.26. Their new baseline at the end of antibiotics was calculated at 1.28, giving them a new to old well baseline of 0.94. A 10% increase above their sick baseline was achieved on days 4 and 6, but this was not a sustained effort.

Overall, 22 of the 25 subjects (88%) had at least 1 day with a 10% improvement over the course of their antibiotics. Of those 22 subjects, 15 (69%) sustained this response. Of these 15 subjects, 14 of them never dipped below this 10% cutoff again during their intravenous antibiotic course. One patient dropped temporarily below their 10% cutoff but then increased over this threshold and sustained that increase. The remaining seven subjects did have a 10% increase at some point in their intravenous antibiotic course, but this was not sustained over their final readings. Typical FEV1 graphs for those with a sustainable increase and those without a sustainable FEV1 increase are shown in Figures 1 and 2.

Of the 15 subjects who met criteria for a sustainable increase, it took an average of 5.2 days after day 1 to achieve this 10% increase. This means that for the average patient, day 6 (rounded down from 6.2) was the first day where their FEV1 measurement was at least 10% greater than the day 1 measurement. The standard deviation for this 10% increase was 4.45 days.

**Figure 3.**
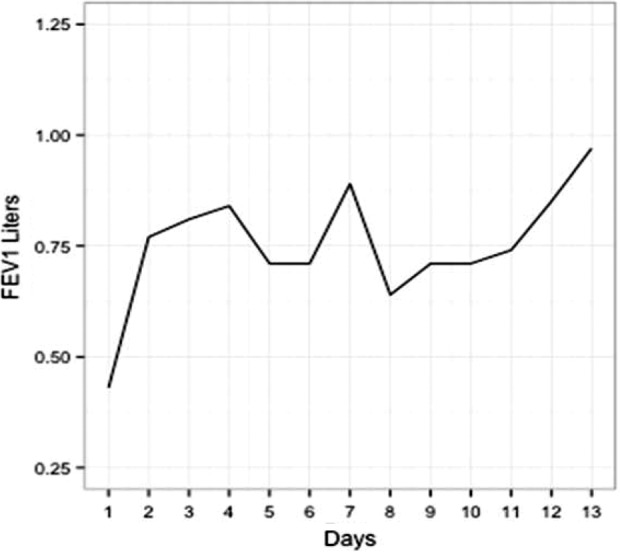
FEV1 graph of patient who did not sustainably maintain within 5% of their final baseline. This subject had a prior well baseline FEV1 of 1.13 (48% predicted) and initially presented with an FEV1 of 0.43. A 10% sustainable increase was seen on day 2. The patients new well baseline was 0.85, indicating they did not achieve their prior well baseline at the end of antibiotics. This patient also did not achieve a sustainable 5% increase in their FEV1 prior to their final three readings. They had an initial increase, and then dropped below the 5% threshold again before rebounding at the end. This highlights variability within patients’ curves in FEV1 on a day to day basis.

The second primary end point was the time to a new baseline FEV1 while on intravenous antibiotics. For all 25 subjects, the average time to a new baseline was on day 6, with a standard deviation of 4.7 days. Of note, only 16 of these 25 patients maintained an FEV1 value within 5% of their new baseline, once it was achieved through the rest of the course of their antibiotics. The other nine subjects achieved a value within 5% of their final new baseline but did not sustain it and dropped below this value again during the course of intravenous antibiotics before going up at the end to their new baseline. An example of one of these nine patients who did not sustain an FEV1 value within 5% of their final baseline is shown in Figure 3.

Those subjects who increased their FEV1 by at least 10% and sustained it were analyzed separately to see when they achieved their new baseline. For these 15 subjects, it took an average of 6.6 days after day 1 to get within 5% of their final baseline. The standard deviation to reach their new baseline was 4.8 days. This would put them at about day 8 when their new baseline was achieved, with a variation between day 3 and day 13.

When the prior well baseline FEV1 was used to see if it would influence whether the subject would have a sustainable increase in their FEV1, neither using FEV1% as a continuous predictor nor using it as a categorical predictor based on whether it was above or below 50% predicted yielded a significant result. The results from these two univariate models are found in Table 2.

**Table 2.** Results of univariate logistic regression models.^a^

Predictor type	Odds ratio	95% confidence interval		*p* Value
Continuous	1.01	0.965	1.054	0.755
Categorical	2.25	0.449	12.404	0.326

^a^Both of these models predict the probability of a subject sustainably increasing their FEV1 based on their baseline percent FEV1. The first model used continuous baseline percent FEV1 as the predictor. The second model used a categorical variable to designate being above or below the median in baseline percent FEV1 as the predictor.

The ratio of the new baseline to their prior well baseline was 0.96, indicating that average subjects did not get back to their prior well baseline. Within this analysis, 9 of 25 subjects (36%) exceeded their prior well baseline, while 16 of 25 (64%) did not achieve their prior well baseline. The standard deviation for the ratio of 0.96 was 0.14.

The ratio of their sick baseline, defined as their FEV1 on day 1, with their prior well baseline was 0.79, with all subjects having a ratio of less than 1. The standard deviation was 0.18.

The CFQ-R results for the analysis at the beginning and end of intravenous antibiotics are shown in Table 3. As seen, the CFQ-R was broken down into its respective categories. The four areas that showed a statistically significant change in the positive direction from the beginning to end of antibiotics were health perceptions, respiratory, vitality, and physical function subscales. The other eight categories showed no difference.

**Table 3.** CFQ-R before and after intravenous antibiotics.^a^

	First CFQ-R scores		Last CFQ-R scores		*p* Value
Section	Mean	Median	Mean	Median	
Physical	37.319	33.333	47.826	41.667	**0.028**
Vitality	52.174	50.000	57.246	58.333	**0.040**
Emotion	67.536	66.667	69.275	73.333	0.678
Eat	80.193	100.000	84.541	100.000	0.479
Treatment burden	36.232	33.333	35.749	33.333	0.681
Health perceptions	58.937	55.556	45.894	44.444	**0.010**
Social	43.237	44.444	43.478	44.444	1.000
Body	70.531	66.667	75.845	77.778	0.114
Role	54.710	58.333	51.812	50.000	0.252
Weight	72.464	100.000	79.710	100.000	0.343
Respiratory	44.203	38.889	60.386	66.667	**0.009**
Digestion	83.575	88.889	82.609	88.889	0.722

CFQ-R: Cystic Fibrosis Questionnaire-Revised.

^a^ *p* Values bolded for significance.

## Discussion

As stated, no adequate trials exist to definitively answer the question of length of IV antibiotics during an acute exacerbation of CF. Several observational trials have been undertaken to see if the following pulmonary function testing could help answer this question. The first of these was published by Redding et al., where 17 children had pulmonary function monitored every other day for 14 days of inpatient intravenous antibiotics.^15^ In this study, there was not a clear optimal length of antibiotics as there was variability in response times as reflected in pulmonary function. Similar results were seen in a retrospective study by Rosenberg and Schramm.^16^ In contrast, a retrospective study by Collaco et al. suggested that antibiotics could be potentially shortened as most improvements in FEV1 occur after 7–10 days of therapy.^17^

The results for this study could be interpreted in several ways. The first would be to focus on the averages of FEV1 increase and time to sustainability. The two numbers to quote would be to expect a 10% increase at day 6, and that patients who increased their FEV1 would plateau at day 8. One would be tempted to cut down antibiotic lengths from 14 days based on this data.

This, however, would not tell the whole story. In those subjects who did sustain a 10% increase, the wide standard deviation of 4.45 days around the mean of 5.2 days would argue against using 5 or 6 days as a meaningful decision point. In looking at the time to a sustainable increase as a potential outcome measure to guide antibiotic length, similar issues arise. A significant portion of subjects, 36%, never reached a sustainable measurement of FEV1 within 5% of their ultimate baseline. This would argue that in an individual patient, FEV1 values fluctuated significantly during their course of intravenous antibiotics, too much to be able to draw conclusions about antibiotic length.

For those that did meet a sustainable threshold within 5% of their final new baseline, they did so on day 8 (mean), which would again appear to be a reasonable number to target. However, again the standard deviation of 4.8 days is so wide that it would be inadvisable to target treatment for all patients for a set length of time.

Using a patients’ baseline well lung function as a predictor for response to intravenous antibiotics also yielded disappointing results. There was also a wide range of presentations of initial sick FEV1 to the prior well baseline. Both of these results speak to the heterogeneity of the population being treated and the fact that it would seem imprudent to recommend similar lengths of antibiotics to this diverse population.

The results, however, are not entirely surprising and are in line with Redding et al. prospective study. CF subjects are colonized with a variety of different bacteria, all of which have variable sensitivities. Subjects with CF also present at different time courses in their exacerbations. There is also a wide range of underlying causes for exacerbations, with adherence to therapy, viral infections, dehydration, or other causes, all playing variable roles.

Given these data, it is our conclusion that the standard of care of 14 days for an acute exacerbation is reasonable, with flexibility for shorter or longer courses as clinically indicated. We know from Elborn et al. that treating subjects without symptoms at regular intervals yielded no difference in outcomes compared to those treated only for symptoms.^20^ A recent retrospective paper by Waters et al. also highlighted the heterogeneity to response to intravenous antibiotics and demonstrated that in a subset of subjects continued improvement in lung function continued after 14 days.^21^

This flexibility would help personalize care for these patients. Unfortunately, the main way to personalize treatment at the current time is based on clinical acumen and experience and not other objective measures. One tool that could help would be utilization of the CF Respiratory Symptom Diary-Chronic Respiratory Infection Symptom Scale (CFRSD-CRISS). In our results, the CFQ-R performed poorly from the beginning to the end of the exacerbation. However, data from the standardized treatment of pulmonary exacerbation study trial recently showed a correlation in the CFRSD-CRISS questionnaire with the beginning and end of intravenous antibiotic treatment.^22^ This could be a potential tool to aid practitioners considering stopping or continuing antibiotics.

There were limitations to the study. The total number of patients was small. It is possible that a bigger sample would yield significant correlations and predictions. It was also largely female. On the whole, however, the population was typical for a CF panel and spanned the typical spectrum of levels of illness seen in CF. In addition, treatment was not standardized across centers.

## Conclusion

In summary, following daily FEV1 during an acute exacerbation can be helpful and give subjects a measure of speed of recovery. It did not, however, show a clear rate of increase and plateau in a cohort as a whole. Given this information, overall variation in treatment length is likely a good thing given the heterogeneity of the population and use of other treatment modalities during an exacerbation. FEV1 should be used in conjunction with clinical signs and symptoms to make a decision of when to start or stop intravenous antibiotics in an individual patient.
